# Downregulated ADAMTS1 Incorporating A2M Contributes to Tumorigenesis and Alters Tumor Immune Microenvironment in Lung Adenocarcinoma

**DOI:** 10.3390/biology11050760

**Published:** 2022-05-16

**Authors:** Hsiao-Chen Lee, Chao-Yuan Chang, Yung-Chi Huang, Kuan-Li Wu, Hung-Hsing Chiang, Yung-Yun Chang, Lian-Xiu Liu, Jen-Yu Hung, Ya-Ling Hsu, Yu-Yuan Wu, Ying-Ming Tsai

**Affiliations:** 1 Graduate Institute of Medicine, College of Medicine, Kaohsiung Medical University, Kaohsiung 807, Taiwan; hc890131@gmail.com (H.-C.L.); chaoyuah@kmu.edu.tw (C.-Y.C.); beryl1992@gmail.com (Y.-C.H.); 980448kmuh@gmail.com (K.-L.W.); ji394122@gmail.com (L.-X.L.); jyhung@kmu.edu.tw (J.-Y.H.); yainghsu@kmu.edu.tw (Y.-L.H.); 2 Division of Plastic Surgery, Department of Surgery, Kaohsiung Medical University Hospital, Kaohsiung Medical University, Kaohsiung 807, Taiwan; 3 Department of Anatomy, Kaohsiung Medical University, Kaohsiung 807, Taiwan; 4 Division of Pulmonary and Critical Care Medicine, Kaohsiung Medical University Hospital, Kaohsiung 807, Taiwan; cyy807@gmail.com; 5 Division of Thoracic Surgery, Department of Surgery, Kaohsiung Medical University Hospital, Kaohsiung Medical University, Kaohsiung 807, Taiwan; shiiiiidae@gmail.com; 6 Division of General Medicine, Kaohsiung Medical University Hospital, Kaohsiung 807, Taiwan; 7 Department of Internal Medicine, Kaohsiung Municipal Ta-Tung Hospital, Kaohsiung 807, Taiwan; 8 Department of Medical Research, Kaohsiung Medical University Hospital, Kaohsiung 807, Taiwan; 9 Drug Development and Value Creation Research Center, Kaohsiung Medical University, Kaohsiung 807, Taiwan; 10 School of Medicine, College of Medicine, Kaohsiung Medical University, Kaohsiung 807, Taiwan; fred901229@gmail.com

**Keywords:** A2M, ADAMTS1, EMT, immunity, LUAD, metastasis

## Abstract

**Simple Summary:**

Lung cancer is the most dreadful cancer type and has the worst cancer-related clinical outcomes. This study used specimens from the in-house lung cancer cohort and public cohort to verify the roles of downregulated ADAMTS1, a protease remodeling extracellular matrix, to facilitate cancer promotion and progress. Based on the clinical specimens, cell and animal study with the aid of the public databases, we concluded that downregulated expression of ADAMTS1 might promote tumor progression and metastasis and modify the tumor microenvironment in lung cancer. Further investigation would be required for its application in treating lung cancer.

**Abstract:**

Lung adenocarcinoma (LUAD) still holds the most dreadful clinical outcomes worldwide. Despite advanced treatment strategies, there are still some unmet needs. Next-generation sequencing of large-scale cancer genomics discovery projects combined with bioinformatics provides the opportunity to take a step forward in meeting clinical conditions. Based on in-house and The Cancer Genome Atlas (TCGA) cohorts, the results showed decreased levels of ADAMTS1 conferred poor survival compared with normal parts. Gene set enrichment analyses (GSEA) indicated the negative correlation between ADAMTS1 and the potential roles of epithelial–mesenchymal transition (EMT), metastasis, and poor prognosis in LUAD patients. With the knockdown of ADAMTS1, A549 lung cancer cells exhibited more aggressive behaviors such as EMT and increased migration, resulting in cancer metastasis in a mouse model. The pathway interaction network disclosed the linkage of downregulated α2-macroglobulin (A2M), which regulates EMT and metastasis. Furthermore, immune components analysis indicated a positive relationship between ADAMTS1 and the infiltrating levels of multiple immune cells, especially anticancer CD4^+^ T cells in LUAD. Notably, ADAMTS1 expression was also inversely correlated with the accumulation of immunosuppressive myeloid-derived suppressor cells and regulatory T cells, implying the downregulated ADAMTS1 mediated immune adjustment to fit the tumor survival disadvantages in LUAD patients. In conclusion, our study indicates that ADAMTS1 interacts with A2M in regulating EMT and metastasis in LUAD. Additionally, ADAMTS1 contributes to poor prognosis and immune infiltration in LUAD patients

## 1. Introduction

According to Global Cancer Statistics 2020, lung cancer is still the leading cause of cancer-related mortality and the second most diagnosed cancer among all cancer types worldwide [1]. Among the two major pathologic categories of lung cancer, non-small cell lung cancer (NSCLC) and small cell lung cancer, NSCLC accounts for 85% and is often diagnosed at an advanced stage. The most common subtype of NSCLC is lung adenocarcinoma (LUAD). Even with multiple therapeutic strategies, the 5-year survival is around 10–20% in the patients with stage IV LUAD [1,2], conferring a substantial medical and economic burden. Therefore, a novel therapeutic strategy is desired to improve patient outcomes.

In the advanced stage, cancer cells can remodel the host tissue microenvironment (TME) through extracellular matrix (ECM) protease to destroy the normal structure of ECM [3]. This aberrant remodeling process plays a crucial role in cancer development and invasion [4]. A disintegrin and metalloprotease with thrombospondin motifs (ADAMTS) family is a group of metalloproteases that polymerize or catalyze ECM [5]. ADAMTS1, a member of the ADAMTS family, remodels chondroitin sulfated proteoglycans and collagen by catalyzing proteoglycan degradation [6]. It can also inhibit angiogenesis by sequestering vascular epithelial growth factor (VEGF) in physiological status [7,8]. Moreover, ADAMTS1 is widely distributed across various tissues or organs to maintain ECM metabolism, including old protein degradation and new protein formation. Therefore, dysregulation of ADAMTS1 has been shown to contribute to various pathological processes of human diseases [9,10]. Many studies have shown the dysregulated activity of ADAMTS1 in tumorigenesis. ADAMTS1 gene is differentially expressed low in various cancer types, including NSCLC, prostate, liver, colorectal, and breast cancers [7,11]. Epigenetic modulation of low ADAMTS1 expression through promoter hypermethylation has also been studied in prostate, colorectal, and lung cancers [7]. However, the role of ADAMTS1 downregulation in lung cancer remains unknown.

ECM is a basic constituent of all tissues and acts as a highly dynamic structure in interactions within a microenvironment, including cancer cell–cancer cell interaction and cancer–non-malignant cell interaction. In addition, the turnover of ECM, such as collagen degradation, is associated with the change of anticancer immunity of TME [12,13]. The current study aimed to investigate the role of ADAMTS1 on cancer progression and immunity of TME. We found that the expression of ADAMTS1 was low in the primary tumors from our patients and public databases. Through next-generation sequencing (NGS), bioinformatic technologies, and functional assays, the ADAMTS1 in advanced LUAD might play a role in the tumor progression and metastasis by changing the host microenvironment. Herein, ADAMTS1 provides an alternative target as a biomarker or therapeutic focus.

## 2. Materials and Methods

### 2.1. Cell Lines

Human lung adenocarcinoma cell line H2087 (CRL-5922™) and A549 cells were obtained from the American Type Culture Collection (ATCC, Manassas, VA, USA) and cultured in RPMI-1640 (for H2087) or F-12K Medium (for A549) supplemented with 10% fetal bovine serum (FBS), 100 U/mL penicillin and 100 μg/mL streptomycin (Thermo Fisher Scientific, Boston, MA, USA). H2087 and A549 cells were authenticated by a short tandem repeat (Promega, Madison, WI, USA) and detected to be negative for mycoplasma contamination by the MycoAlert™ mycoplasma detection kit (Lonza, Switzerland) every 3 months. Lung cancer cells were cultured under normoxic (20% O_2_), hypoxic (2% O_2_) conditions for 24 h (short-term hypoxia) or 100 days (long-term hypoxia), respectively.

### 2.2. Bioinformatics

The differential expressions of *ADAMTS1* and other selected mRNAs between *LUAD* and normal lung tissue were extracted from the Oncomine database (http://www.oncomine.org (accessed on 1 September 2021), Compendia biosciences, Ann Arbor, MI, USA) [14] or The Cancer Genome Atlas (TCGA) using UALCAN website (http://ualcan.path.uab.edu/ (accessed on 1 September 2021)) [15]. The criteria for the significant mRNAs in the analysis were a fold change (tumor/normal) of expression level > 2 and a *p*-value < 0.05, which was calculated using the Oncomine or UALCAN website. The KM plotter database (http://kmplot.com/analysis/ (accessed on 1 September 2021)) [16] was used to analyze the association of the mRNA expression with overall survival (OS), time to first progression (FP), and post-progression survival (PPS). Patients were divided into 2 groups by median value, which was computed with median survival. The hazard ratios (HR) with 95% confidence intervals (CI) and *p*-values were extracted from the KM plotter webpage and considered significant with *p*-values < 0.05. The functional states of the selected genes of interest in LUAD were assessed by the CancerSEA website (http://biocc.hrbmu.edu.cn/CancerSEA/ (accessed on 29 March 2022)) [17]. GSVA (Gene Set Variation Analysis) score of ADAMTS1 and A2M was calculated by the GSCAL website (http://bioinfo.life.hust.edu.cn/GSCA (accessed on 11 January 2022)) [18]. The Pathway Commons was utilized to acquire the protein interaction (https://www.pathwaycommons.org/ (accessed on 1 September 2021)) [19].

### 2.3. NGS and Quantitative Real-Time Polymerase Chain Reaction (qRT-PCR)

The pairs of adjacent non-tumor lungs and tumors were harvested from the Division of Thoracic Surgery and Division of Pulmonary and Critical Care Medicine, Kaohsiung Medical University Hospital (Kaohsiung, Taiwan, KMUH-IRB-20130054; KMUH-IRB-20180023). The RNA sequencing for the pairs of LUAD and lung normal tissue was performed by the Welgene biotechnology company (Taipei, Taiwan). The criteria for differentially expressed mRNA by NGS analysis were fold change > 2 and fragments per kilobase million (FPKM) > 0.3.

Total RNA was isolated from cells using the TRIzol Reagent (Life Technologies, Carlsbad, CA, USA), and cDNA was reverse transcribed using reverse transcriptase kits (Takara, Shiga, Japan). RNA levels were detected using real-time analysis with SYBR Green on a QuantStudio 5 machine (Thermo Scientific, CA, USA). The relative expression levels of the specific mRNAs were normalized to glyceraldehyde 3-phosphate dehydrogenase (GAPDH). The relative standard method (2^−ΔΔCt^) was used to calculate relative RNA expression. The following primers were used: ADAMTS1 (forward, 5′- CGGAAGTGACCTCCAATGCT-3′ and reverse, 5′-CTGCTCGGATCACACACAGT-3′), and GAPDH (forward, 5′-TTCACCACCATGGAGAAGGC-3′ and reverse, 5′-GGCATGGACTGTGGTCATGA-3′).

### 2.4. Immunoblot

The total protein of A549 cells was extracted using the radio-immunoprecipitation assay (RIPA) (EMD Millipore, Billerica, MA, USA) supplemented by a protease inhibitor cocktail (Sigma-Aldrich, St. Louis, MO, USA). An equal volume of total protein was denatured by heating and then separated by a sodium dodecyl-sulfate polyacrylamide gel electrophoresis. Proteins in the gel were transferred onto polyvinylidene difluoride membranes (EMD Millipore) by electroblotting, which was probed with various primary antibodies overnight after blocking in 5% nonfat dry milk/TBST, followed by incubation with horseradish peroxidase (HRP)-conjugated secondary antibodies (Cell-Signaling Technology, Danvers, MA, USA). The signal of the specific protein was detected using a chemiluminescence kit (EMD Millipore). Primary antibodies used include those against Slug (catalog# 9585) and Snail (catalog#3879) were obtained from Cell Signaling Technology (Carlsbad, CA, USA). Anti-N-cadherin (catalog#610921), E-cadherin (catalog#610182), and Vimentin (catalog#550513) antibodies were purchased from Becton Dickinson biosciences. Anti-α-smooth muscle actin antibody (catalog#A5228) and GAPDH (catalog#MAB374) antibodies were acquired from EMD Millipore. The western blot was quantified by ImageJ. 

### 2.5. Characterization of the Tumor Immune Microenvironment

TIMER (http://timer.cistrome.org/ (accessed on 29 March 2022)) [20] was used to predict the immune profile of LUAD. It is a comprehensive resource including three modules for exploring the association between infiltrating immune cells and genes across various cancer types. We evaluated the association between the *ADAMTS1* expression and the infiltration levels of immune cells in LUAD using the “Immune Association” module. The criteria for the positive or negative correlation between *ADAMTS1* and immune cells was a *p* value < 0.05.

### 2.6. ADAMTS1 Knockdown

Knockdown of ADAMTS1 in A549 cells was performed by using shRNA plasmids (#1, TRCN0000052110, CGAGTGTGCAAAGGAAGTGAA; #2, TRCN0000052112; CCACAGGAACTGGAAGCATAA; #3, TRCN0000052108, GCCTACATGATTACATCATTT) expression system obtained from the National RNAi Core Facility (Taipei, Taiwan). The stable clone of *ADAMTS1* knockdown cells by shRNA (clone ID: TRCN0000052110) was established by the puromycin selection. The knockdown efficacy of *ADAMTS1* shRNA plasmid was determined by qRT-PCR.

### 2.7. Wound Healing Analysis

Control vector and *ADAMTS1*-knockdown A549 cells were seeded onto a 12 well-plate at 100% confluence, and the cell migration ability was determined by measuring the movement of cells into the acellular area created by a sterile tip. The cells at each well were observed at 0 and 24 hours after wound making under a microscope.

### 2.8. Animal Model

Control plasmid transfected and *ADAMTS1*-knockdown A549 cells (1 × 10^6^) were transplanted into nude mice by tail vein injection [21]. Animals were sacrificed on week 12, and the number of tumor nodules in the lungs was counted. The lungs of mice were fixed and embedded in paraffin for histological hematoxylin and eosin staining. The whole field images of lungs were acquired using a TissueFAXS system by TissueFAXS instrument (TissueGnostics, Vienna, Austria). All mice (8 weeks, *n* = 6 for each group) were purchased from the National Laboratory Animal Center Taiwan and housed in a specific pathogen-free environment. All mice were housed in a specific pathogen-free environment. Two experiments were conducted in accordance with the National Institutes of Health Guide for the Care and Use of Laboratory. The animals used were approved by the KMU Animal Care and Use Committee (KMU−108158).

### 2.9. Statistical Analysis

Results were presented as mean ± standard deviation (SD). Multiple group comparisons were calculated by one-way analysis of variance (one-way ANOVA) with a Tukey’s post hoc test used with the assistance of the GraphPad Prism program (9.02 version, Graphpad Software, San Diego, CA, USA). Two tested groups were compared using a Student’s *t*-test. Results were considered statistically significant when the *p*-value was less than 0.05. Pearson correlation and multiple linear regression by R package (estimatr) were used for correlation.

## 3. Results

### 3.1. Lung Tumors Expressed Lower Levels of ADAMTS1

**Figure 1:**
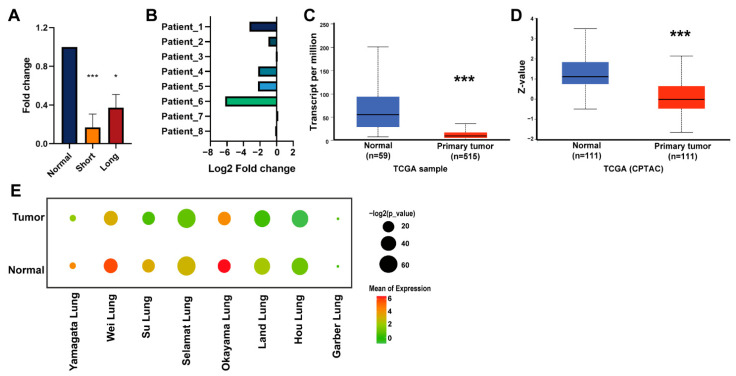
**The expression of *ADAMTS1* in lung adenocarcinoma** (**LUAD**)**.** (**A**) Fold change of *ADAMTS1* expression from the lung cancer cells H2087 cultured in normoxia and hypoxia conditions for 24 h (short) or 100 days (long). The RNA levels of *ADAMTS1* were determined by qRT−PCR. (**B**) Fold change of *ADAMTS1* expression in the paired tumor and normal tissue from eight patients with LUAD (in−house cohort). *ADAMTS1* mRNA (**C**) and protein (**D**) expression in the normal tissue and tumor from TCGA cohort. (**E**) *ADAMTS1* mRNA expression in the normal tissue and tumor of eight datasets from the Oncomine datasets. Data shown represent the mean ± SD (* *p* < 0.05, *** *p* < 0.001).

Hypoxia potentiates malignant potential in cancer. Therefore, we exposed cancer cells to hypoxia conditions, which allowed us to figure out the targets related to aggressive phenotypes [22]. Our results showed that the expression of *ADAMTS1* in H2087 cells was decreased under both short-term hypoxia and long-term hypoxia conditions (Figure 1A). In addition, lower levels of *ADAMTS1* were found in five out of eight LUAD samples compared with those in matched non-tumorous lung tissues (Figure 1B). The downregulation of ADAMTS1 was also proven at both mRNA and protein levels in the TCGA cohort (Figure 1C,D). Similarly, seven out of eight patients in the lung cancer cohort from Oncomine also supported *ADAMTS1* being reduced in lung tumor tissue (Figure 1E). These data suggest that reduced *ADAMTS1* may be associated with lung cancer development.

### 3.2. The Phenotypic Association and Survival Significance of ADAMTS1

**Figure 2:**
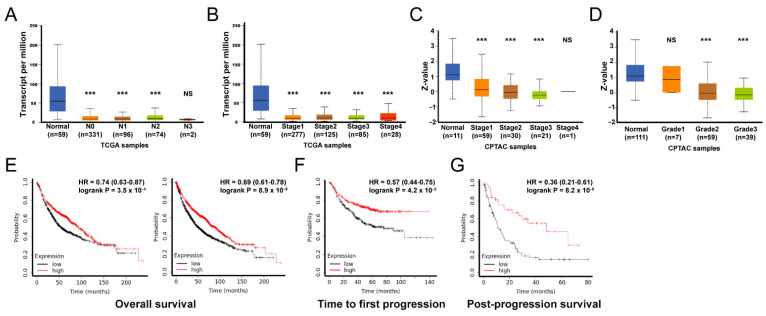
**Tumor characteristics and survival analysis of *ADAMTS1* expression in LUAD.** The mRNA expression of *ADAMTS1* among the tumor nodal metastasis (**A**) and stages (**B**) in the TCGA cohort. The protein expression among the tumor stages (**C**) and grade (**D**) in the CPTAC cohort. Overall survival (OS) (**E**), time to first progression (FP) (**F**), and post-progression survival (PPS) (**G**) with *ADAMTS1* expression level were analyzed using Kaplan–Meier methods in the public microarray and TCGA dataset. (*** *p* < 0.005).

Various clinical features based on the transcriptional level of *ADAMTS1* in 515 samples in the TCGA LUAD cohort were elucidated. The *ADAMTS1* transcriptomic level was significantly lower in tumor parts throughout different extents of lymph node (N0−N3) metastasis and pathological stages (stage I to IV), but were not lymph node metastasis or stage dependent (Figure 2A,B). Similarly, the levels of ADAMTS1 protein were also reduced in tumor tissue across different extents of lymph node metastasis and histologic grades (grade 1 to grade 3) of cancer cells, but were not lymph node metastasis or grade dependent (Figure 2C,D). Next, we assessed the prognostic implication of ADAMTS1 in LUAD patients using the Kaplan–Meier Plotter database. Our results showed that the LUAD patients with downregulated *ADAMTS1* mRNA levels carried unfavorable overall survival (OS), time to first progression (FP), and post-progression survival (PPS) (Figure 2E–G). These results showed that ADAMTS1 is positively correlated with survival in lung cancer patients.

### 3.3. Protein Interaction Network of ADAMTS1

**Figure 3:**
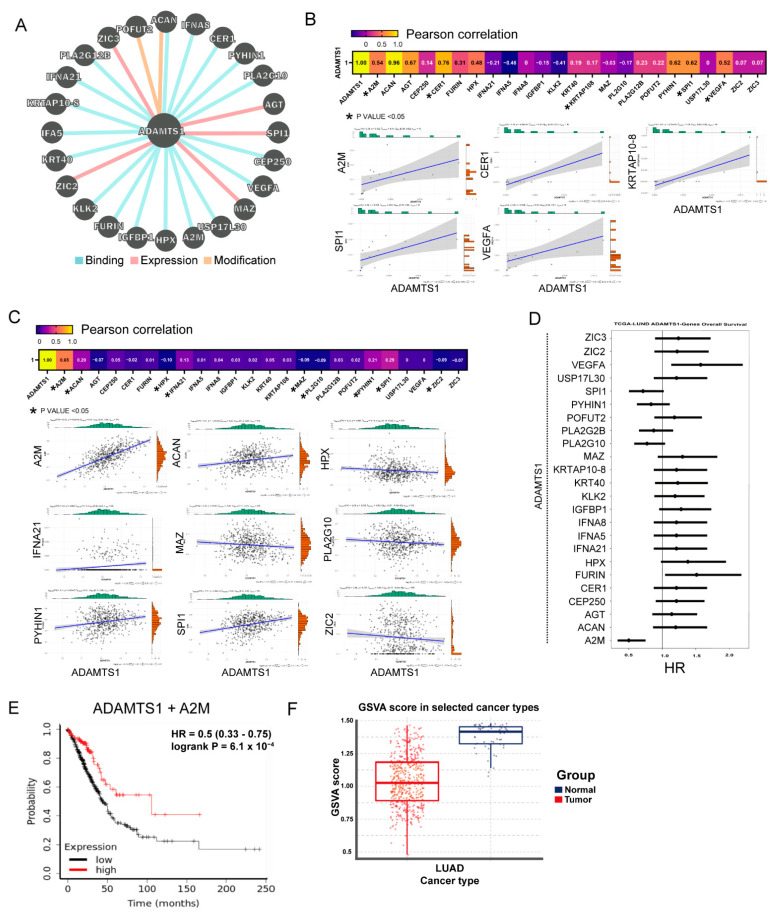
**Interaction network of *ADAMTS1* in LUAD.** (**A**) Interaction network of *ADAMTS1* with 25 other genes analyzed by Pathway Commons. (**B**) Correlation between *ADAMTS1* and these genes in the in-house LUAD patients (**C**) and in the TCGA cohort (**D**). (**E**) Weight analysis of *ADAMTS1* and *A2M* on overall survival (OS) of patients with LUAD. (**F**) The gene set variation analysis (GSVA) analysis of *ADAMTS1* and *A2M* in TCGA datasets.

The Pathway Interaction Database provided the molecular interactions with ADAMTS1. The 24 proteins interacting with ADAMTS1 were predicted based on binding, expression regulation, and modification (Figure 3A). We evaluated the possible cooperation of candidate proteins with ADAMTS1 by assessing the expression correlation in the tumor of in-house and TCGA cohorts. As shown in Figure 3B,C, the expressions of A2M, CER1, KRTAP10-8, VEGFA, and SPI1 exhibited significant correlations with ADAMTS1 in our in-house tissue bank (Figure 3B), while A2M, ACAN, HPX, IFNA21, MAZ, PLA2G10, PYHIN1, SPI1, and ZIC2 were significantly correlated with ADAMTS1 expression in the TCGA LUAD cohort (Figure 3C). In addition, when inputting specific protein–ADAMTS1 with 1:1 weight into the survival analysis, it was revealed that higher expression of A2M-ADAMTS1 offered a survival advantage (Figure 3D). Additionally, the multiple linear regression suggested that the strongest correlation with ADAMTS1 was A2M (Appendix Table A1). Moreover, GSVA analysis showed that the GSVA score of ADAMTS1 plus A2M was lower in tumors than in normal parts in the TCGA LUAD cohort (Figure 3F). The results suggest that co-existing ADAMTS1 and A2M is a positive prognostic factor for patients with LUAD.

### 3.4. The Impact of A2M in Patients with LUAD

**Figure 4:**
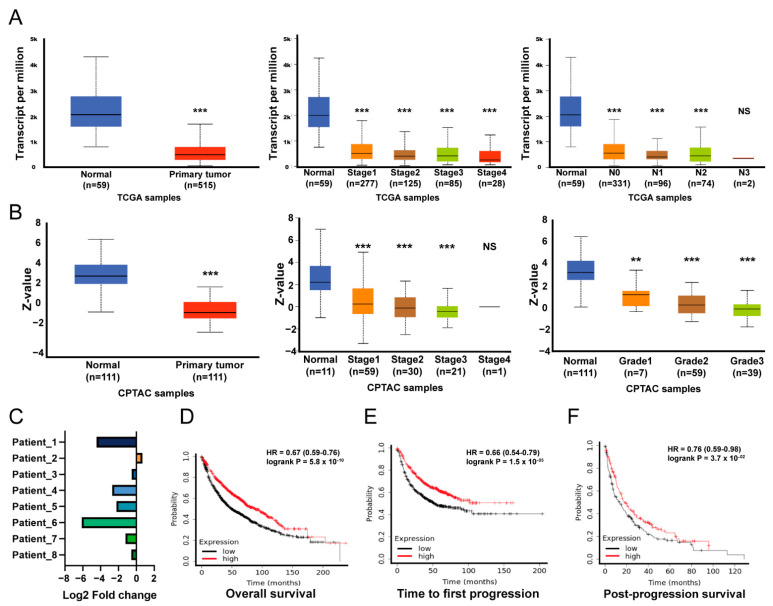
**Tumor characteristics of A2M expression and survival analysis of ADAMTS1-A2M expression in LUAD.** (**A**) The expression of *A2M* mRNA among the normal tissue and tumor (left), the tumor stages (middle), and the nodal metastasis (right) in the TCGA cohort. (**B**) The expression of A2M protein expression among the normal tissue and tumor (left), the tumor stages (middle), and the tumor grades (right) in the CPTAC cohort. (**C**) The level of *A2M* in the tumor and normal tissue from eight patients with LUAD (in-house cohort). Correlation of OS (**D**), FP (**E**), and PPS (**F**) with *A2M* expression in patients with LUAD. (NS not significant, ** *p* < 0.01, *** *p* < 0.005).

Next, we explored the prognostic role of A2M in LUAD patients. The expression of *A2M* mRNA was low in tumor parts compared with normal parts, and so were lymph node metastasis and tumor stages. However, they were not lymph node metastasis nor stage dependent (Figure 4A). In addition, the expression levels of the A2M protein were low in tumor parts compared to normal ones. The A2M protein levels were lower based on stages and tumor histologic grades, but were not stage or grade dependent (Figure 4B). The expression of A2M also decreased in five out of the eight LUAD patients in the in-house cohort (Figure 4C). The survival analysis revealed a shorter OS, FP, and PPS when expressing low-level *A2M* in LUAD patients (Figure 4D–F). It is suggested that decreased A2M expression was a strong predictor of worse prognoses among LUAD patients.

### 3.5. GSEA Analysis of ADAMTS1

**Figure 5:**
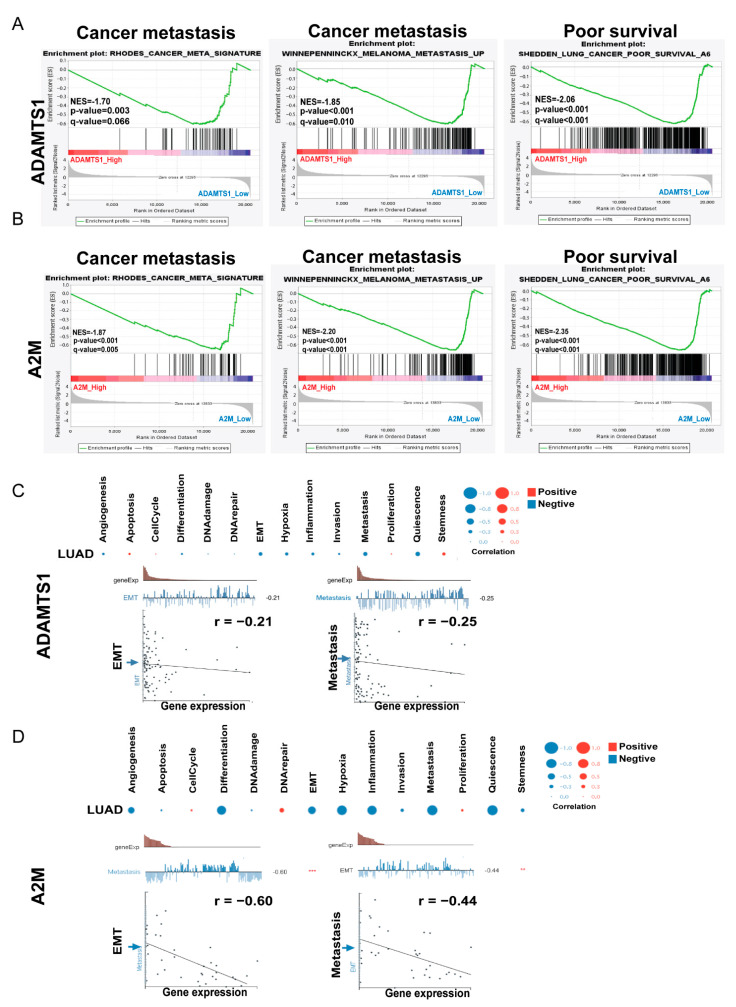
**Functional states associated with *ADAMTS1* and *A2M*.** (**A**) Functional analyses of cancer metastasis and poor survival via gene set enrichment analysis (GSEA) in TCGA LUAD patients with high or low *ADAMTS1* levels (*p* < 0.05). (**B**) Functional analyses of cancer metastasis and poor survival via GSEA analysis in TCGA LUAD patients with high or low *A2M* levels (*p* < 0.05). (**C**) The analysis of functional states of *ADAMTS1* using CancerSEA. (*p* < 0.05). (**D**) The analysis of functional states of *A2M* using CancerSEA (*p* < 0.05). NES, normalized enrichment score. ** *p* < 0.01, *** *p* < 0.001.

To gain insight into the functional roles of *ADAMTS1* and *A2M* in LUAD, we performed two gene enrichment analyses, GSEA and CancerSEA. The GSEA results suggested that decreased *ADAMTS1* in lung cancer was associated with poor survival and cancer metastasis (Figure 5A). Consistent with *ADAMTS1*, enrichment analysis also revealed that tumors with lower levels of *A2M* were strongly associated with poor survival and cancer metastasis (Figure 5B). In addition, CancerSEA analysis, which was empowered to evaluate the functional assays of a selected gene, showed a strong correlation between *ADAMTS1* and *A2M*, with phenotype changes contributing to cancer spreading, including EMT, metastasis, and quiescence in lung cancer (Figure 5C,D). These bioinformatics data suggest that decreased *ADAMTS1* and *A2M* levels are associated with more aggressive phenotype in LUAD.

### 3.6. The Role of ADAMTS1 in the Tumor Microenvironment

**Figure 6:**
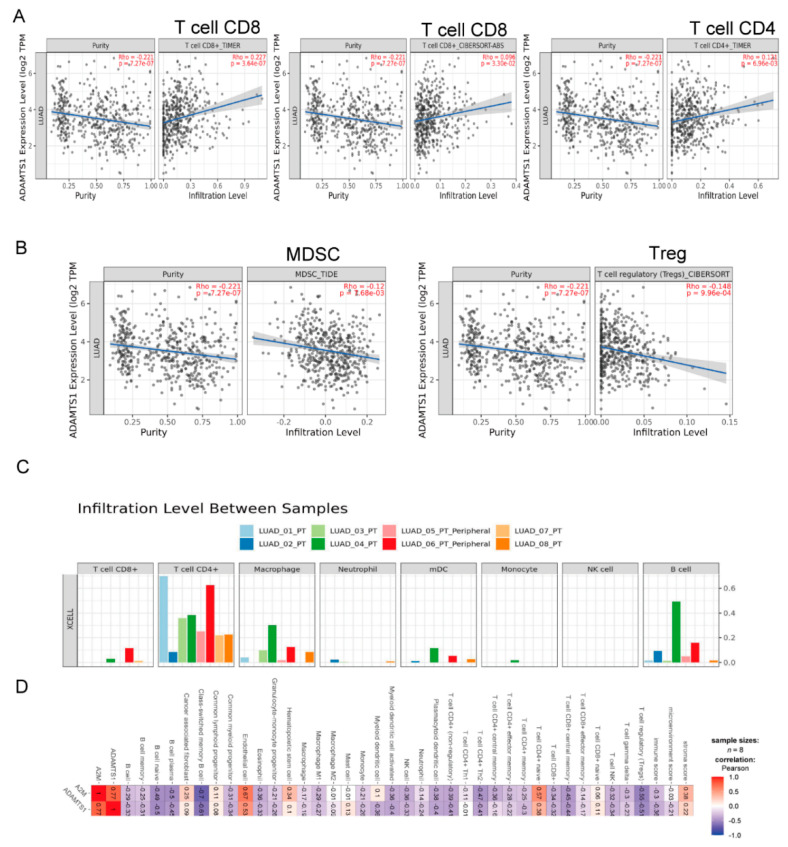
**Correlation of immune or stroma score with *ADAMTS1* and *A2M* in LUAD.** Positive (**A**) and negative (**B**) correlation of immune cell population with *ADAMTS1*. (**C**) The immune cells population of the LUAD patients from our 8 in−house LUAD patients. (**D**) The correlation of the immune population with the level of *ADAMTS1* in 8 in−house LUAD patients.

Because *ADAMTS1* is an extracellular protease, which is required for a balanced immune cell repertoire and tumor inflammatory response [23], we assessed the role of *ADAMTS1* in the tumor immune microenvironment using the Timer 2.0 website. Our results showed that CD8^+^ and CD4^+^ T cells were the main populations of immune cells affected by *ADAMTS1* expression. Among them, CD4^+^ and CD8^+^ T cells exhibited a positive correlation with the level of *ADAMTS1* (Figure 6A). Conversely, Treg and MDSC were the immune populations with a significantly negative correlation with the expression of *ADAMTS1* (Figure 6B). We further assessed the immune landscape of tumors in the in-house database using Timer 2.0 (Figure 6C). Results showed that CD4^+^ T cells and hematopoietic stem cells have a positive correlation with the level of *ADAMTS1*. Interestingly, most types of immune cells, including Treg, have a negative correlation with the expression of *ADAMTS1* (Figure 6D).

### 3.7. Knockdown of ATAMDS1 Promotes Cancer Migration, EMT, and Metastasis In Vitro and In Vivo Models

**Figure 7:**
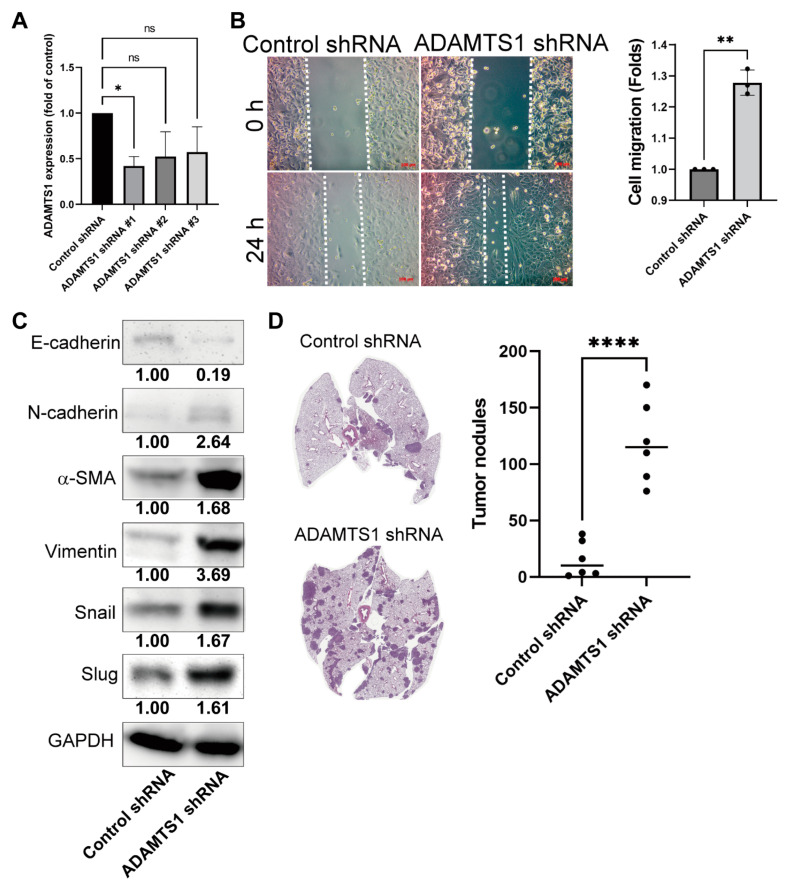
**Functional analysis of ADAMTS1 in lung adenocarcinoma.** (**A**) The knockdown efficacy of shRNA transfection. (**B**) Cell migration of *ADAMTS1* knockdown A549 cells, as determined by wound-healing. (**C**) EMT markers in the *ADAMTS1* knockdown A549 cells. The uncropped western blot figures were presented in Figure S1. (**D**) Tumor metastasis in mice lung after injection of control plasmid transfected and *ADAMTS1*-knockdown A549 cells. A549 cells were transfected with a control vector or *ADAMTS1* shRNA plasmid and selected by puromycin. The cells were implanted into nude mice by tail vein injection, and the tumor nodules were counted after 21 days of inoculation. The data showed represent the mean ± SD (* *p* < 0.05, ** *p* < 0.01, **** *p* < 0.001). ns, not significant.

To further validate the roles of ADAMTS1 in mediating lung cancer progression and promotion, this study used the shRNA technique to knock down *ADAMTS1* in A549 cells. Because shRNA#1 exhibited the highest efficacy of *ADAMTS1* knockdown in A549 cells, we chose this clone for functional analysis (Figure 7A). The wound-healing assay by control-shRNA and *ADAMTS1*-shRNA showed an enhanced healing process (Figure 7B). Additionally, when *ADAMTS1* was knocked down, the A549 cells shifted from the epithelial (E-cadherin) to the mesenchymal (N-cadherin, α-SMA, Vimentin, Snail, and Slug) phenotype (Figure 7C). Concerning metastasis, the tail vein injection model was used. The *ADAMTS1*-plasmid transfected A549 cells were injected into the nude mice to verify lung cancer metastasis. The result suggested more metastatic nodules occurred in the *ADAMTS1*-shRNA group (Figure 7D). These results imply that the low-level ADAMTS1 mediates a more aggressive phenotype in LUAD.

## 4. Discussion

Although the diagnosis and treatment of LUAD are constantly improving, LUAD is a high-risk disease with a 5-year survival rate of 16% [2], and the potential mechanisms underlying the development and progression of LUAD are still remaining to be determined. Over the past decade, an increasing number of microarray and next-generation sequencing technologies applied in comprehensive genomic atlases, such as TCGA, have been used to explore novel prognostic biomarkers and therapeutic targets in various cancers. Herein, we investigated the clinical significance of ADAMTS1 in LUAD using multiple bioinformatic tools as well as clinical samples. We found that ADAMTS1 could be exploited as a prognostic predictor and therapeutic target due to its effect on the mesenchymal phenotypic transition and cancer immune microenvironment.

The role of ADAMTS1 in oncology has been investigated in various cancers in the past decade. However, controversy regarding cancer and ADAMTS1 exists, and its role is still under debate, since it could act as either a tumor suppressor or an oncogene depending on the cellular context or specific cancers. There is increasingly abundant evidence that the metalloprotease ADAMTS1 is strongly correlated with metastasis of breast cancer [24]. In contrast, ADAMTS1 may have a suppressive activity in tumor cell growth and progression in protease-dependent and -independent manners. ADAMTS1 decreases fibrosarcoma cell proliferation and migration velocity by disrupting HGF/c-MET signaling [25]. In addition, Wang et al. reported that ADAMTS1 inhibits angiogenesis in lung cancer by regulating VEGF expression through a PI3K/AKT inhibition mechanism [26]. A recent study also showed that ADAMTS1 decreases cancer migration by regulating the spatiotemporal dynamics of Cdc42 activity [27]. This discrepancy might be due to cleavage or to an auto-proteolytic mechanism [28]. Our findings revealed that ADAMTS1 was a target of hypoxia, which acts as a pivotal regulator in stimulating the development of an aggressive phenotype in cancer [29,30]. In this study, we found that the expression of *ADAMTS1* dramatically decreased in LUAD patients, who have poor clinical outcomes, supporting the notion that ADAMTS1 holds great promise for the improvement of prognostic prediction in LUAD. Additionally, GSEA analysis showed the enrichment of EMT and metastatic signaling pathways with low *ADAMTS1* expression of lung cancer in the TCGA cohort. Meanwhile, CancerSEA analysis also indicated that *ADAMTS1* expression was negatively associated with metastasis, supporting the suppressive role of ADAMTS1 in LUAD. The in vitro and in vivo functional assays also demonstrated that knockdown of *ADAMTS1* transformed A549 cells to the mesenchymal phenotype, resulting in the enhancement of lung metastasis in the mice model. All of the results above indicate that ADAMTS1 has a tumor suppressive role on LUAD.

As for the downstream target, we suggested that ADAMTS1 decreased cancer development of LUAD by incorporating α2-macroglobulin (A2M). A2M could inactivate a variety of proteases by inhibiting plasmin and kallikrein, and could also act as the carrier protein that binds to various growth factors, hormones, and cytokines such as platelet-derived growth factor, basic fibroblast growth factor, insulin-like growth factor, interleukin and TGF-β1 [31]. A2M is also indicated to impede β-catenin signaling, thus consequently inhibiting the malignant properties of astrocytoma cells [32]. Other tumorigenesis-related signaling pathways, such as PI3K/AKT and microRNA-21, have also been reported as inhibitory targets of A2M [33]. ADAMTS1 has been indicated to be able to form a covalent binding complex with A2M by zinc-binding motif interaction [8]. In our study, we found that the expression of ADAMTS1 had a strong association with the level of A2M in LUAD patients. Similar to the impact of ADAMTS1, patients with lower levels of ADAMTS1 were closely related to a poor prognosis of LUAD. Function enrichment analysis by computational algorithms also showed that downregulated A2M was related to EMT and metastasis in LUAD. However, the interaction between A2M and ADAMTS1 in LUAD has not been fully understood, and it is necessary to explore the molecular mechanism further.

The tumor microenvironment (TME) plays important roles in the immunity, progression, and metastasis of various cancers, including LUAD. The presence of a chronic inflammatory status alters immune cell differentiation and activity, resulting in an imbalance of anti-cancer activity, thus favoring malignant evasion [34]. Myeloid-derived suppressor cells (MDSCs) and regulatory T lymphocytes (Treg) are involved in tumor-associated immunosuppression and associated with poor clinical outcomes in lung cancer patients [35]. MDSCs, a heterogeneous population of pathologically activated myeloid cells, promote angiogenesis, facilitate EMT, enhance cancer stemness capabilities, protect circulating tumor cells from host immunity, and contribute to resistance to immunotherapy [36]. Treg reduces the co-stimulation ability of an antigen-presenting cell or direct inactivation effector T cell by expressing PD ligand 1 or cytotoxic T-lymphocyte-associated protein 4. In addition, both MDSCs and Treg produce various immunosuppressive cytokines or mediators, such as IL-10, TGF-β, and ROS, which can interact with nearby T cells or other immune cells and prevent their anticancer activation of immune cells [37]. ADAMTS1 has been indicated to alter the infiltration of immune cells in B16F1 cancer by increasing CD3^+^ T cells and CD11b^+^ myeloid cells with a decreased CD163 expression in ADAMTS1-deficient mice [23]. In this study, we found that ADAMTS1 influenced the infiltration of immune cells in the lung cancer microenvironment. High ADAMTS1 expression was associated with a high percentage of CD8^+^ T cells and CD4^+^ T cells. In contrast, low ADAMTS1 expression was related to a high percentage of MDSCs and Treg. Among immune cell types, ADAMTS1 had the strongest and most consistent correlation with CD8^+^ T cells. Upregulation of ADAMTS1 may increase T cell immune infiltration and function of lung cancer, thereby improving the disease outcome of LUAD patients.

## 5. Conclusions

In conclusion, our study found ADAMTS1 downregulation accompanied by A2M downregulation could promote the LUAD metastasis by activating EMT and changing the immune microenvironment. This study provides crucial clues to the mechanism of LUAD metastasis. Moreover, as a prognostic indicator, ADAMTS1 and its co-operator A2M could be potential therapeutic targets for patients with LUAD.

## Data Availability

The authors also thanks the websites bellow for data acquisition (all accessed on 1 September 2021):
The CancerSEA website (http://biocc.hrbmu.edu.cn/CancerSEA/). The GSCAL website (http://bioinfo.life.hust.edu.cn/GSCA). The KM plotter database (http://kmplot.com/analysis/). The Oncomine database: http://www.oncomine.org. The Pathway Commons (https://www.pathwaycommons.org/). TIMER (http://timer.cistrome.org/). The UALCAN website (http://ualcan.path.uab.edu/).

## Supplementary Materials

The following supporting information can be downloaded at: https://www.mdpi.com/article/10.3390/biology11050760/s1. The uncropped western blot figures were presented in Figure S1.

## Appendix A

**Table A1:** Multiple linear regression analysis of ADAMTS1 in TCGA-LUAD.

Factors	Coefficient	Standard Error	*p*-Value	95% Confidential Interval
***A2M***	0.713	0.044	***	0.626 to 0.8
***ACAN***	0.096	0.025	***	0.047 to 0.145
***HPX***	−0.040	0.023	0.076	−0.084 to 0.004
***IFNA21***	0.227	0.151	0.134	−0.07 to 0.524
***MAZ***	−0.139	0.056	*	−0.249 to −0.028
***PLA2G10***	−0.111	0.022	***	−0.154 to −0.068
***PYHIN1***	−0.026	0.035	0.458	−0.094 to 0.042
***SPI1***	−0.082	0.046	0.076	−0.172 to 0.008
***ZIC2***	−0.051	0.017	**	−0.084 to −0.018

* *p* < 0.05, ** *p* < 0.01, *** *p* < 0.001.
