# Supplementary material for: Downregulated ADAMTS1 Incorporating A2M Contributes to Tumorigenesis and Alters Tumor Immune Microenvironment in Lung Adenocarcinoma
Source: Biology (Basel). 2022 May 16;11(5):760. doi: 10.3390/biology11050760 (PMC9139094; doi:10.3390/biology11050760)

E-cadherin
BD (610182) 120 kDa

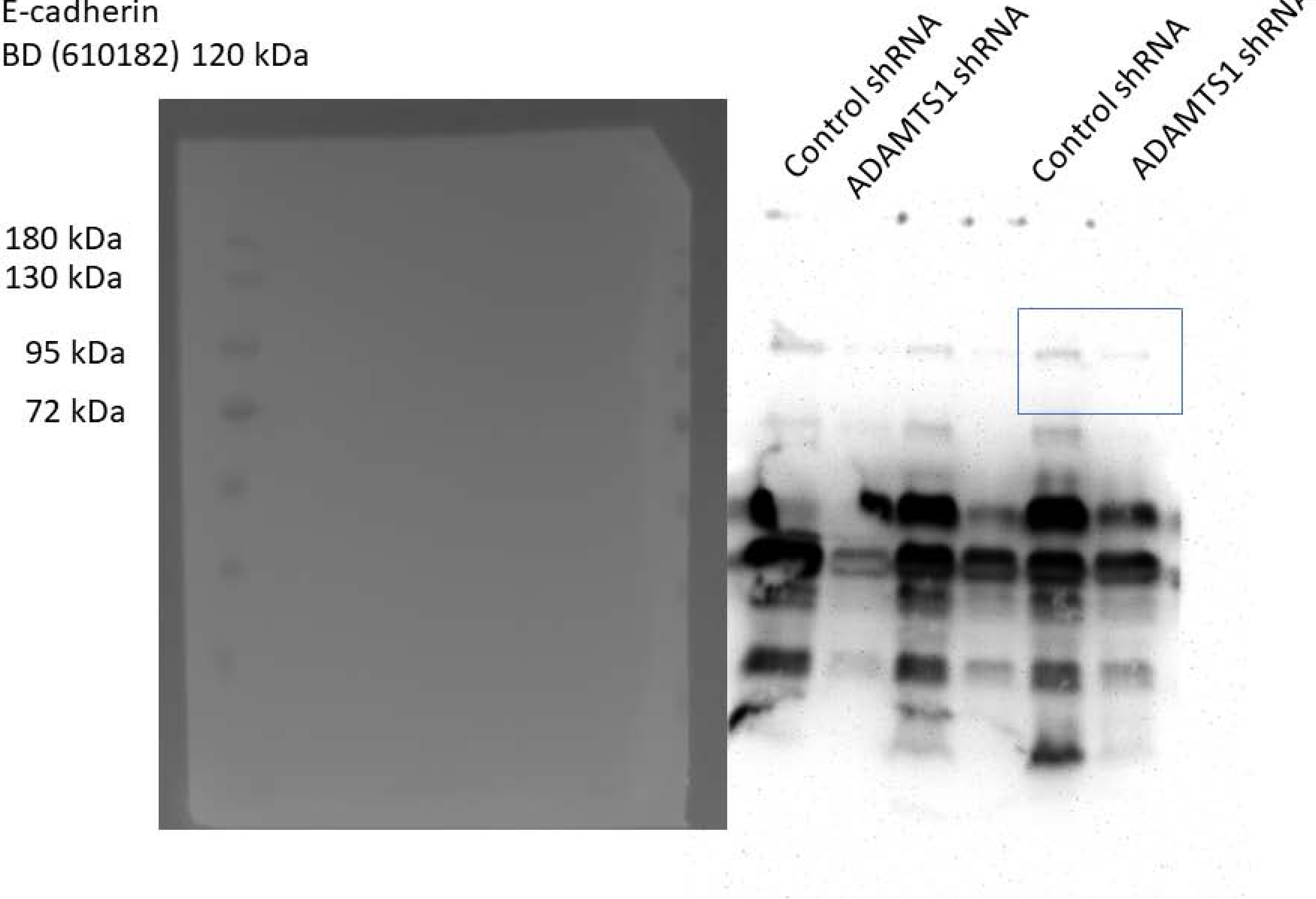

N-cadherin
BD (610921)
130 kDa

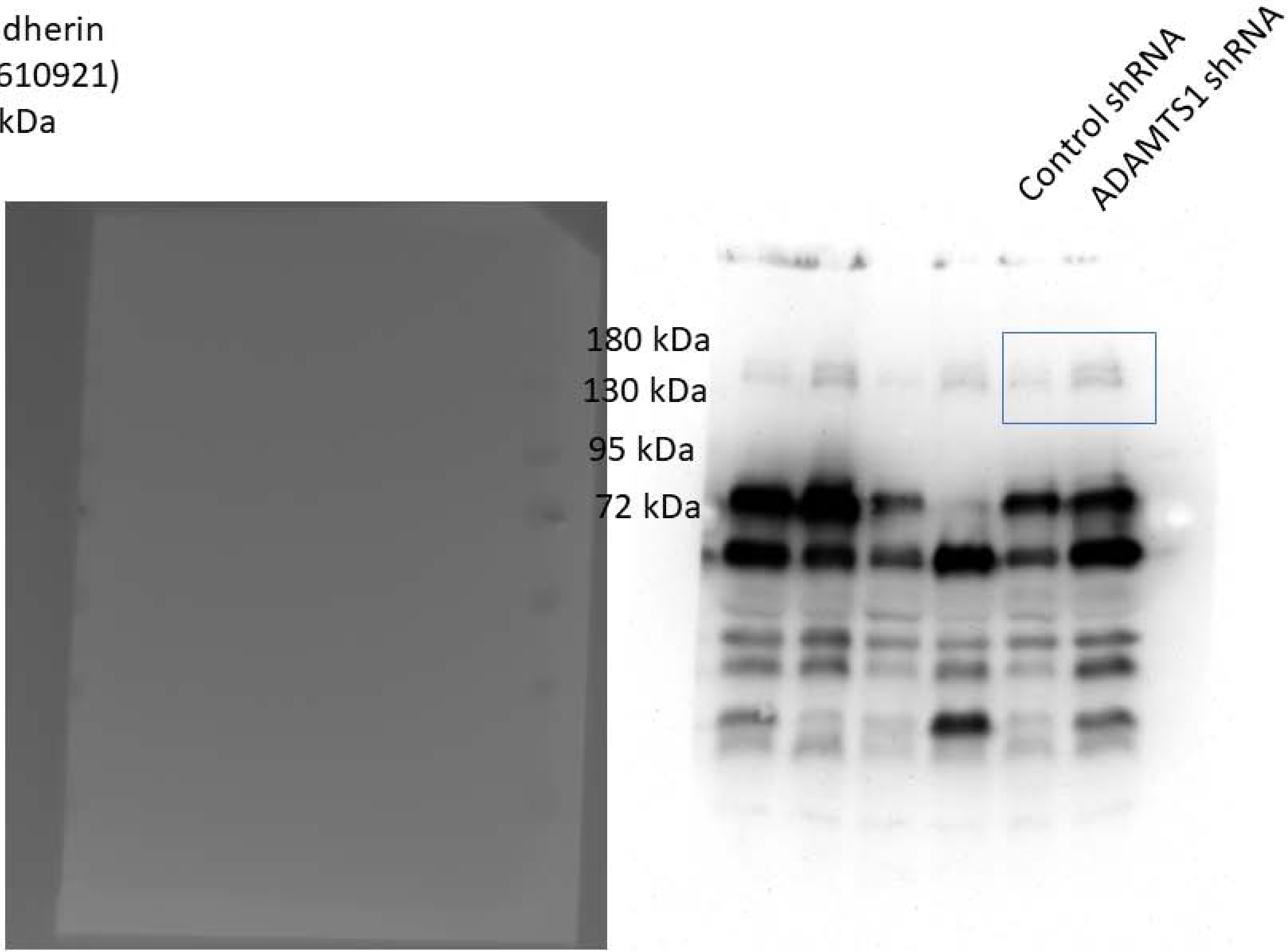

α-SMA
Sigma (A5228)
42 kDa

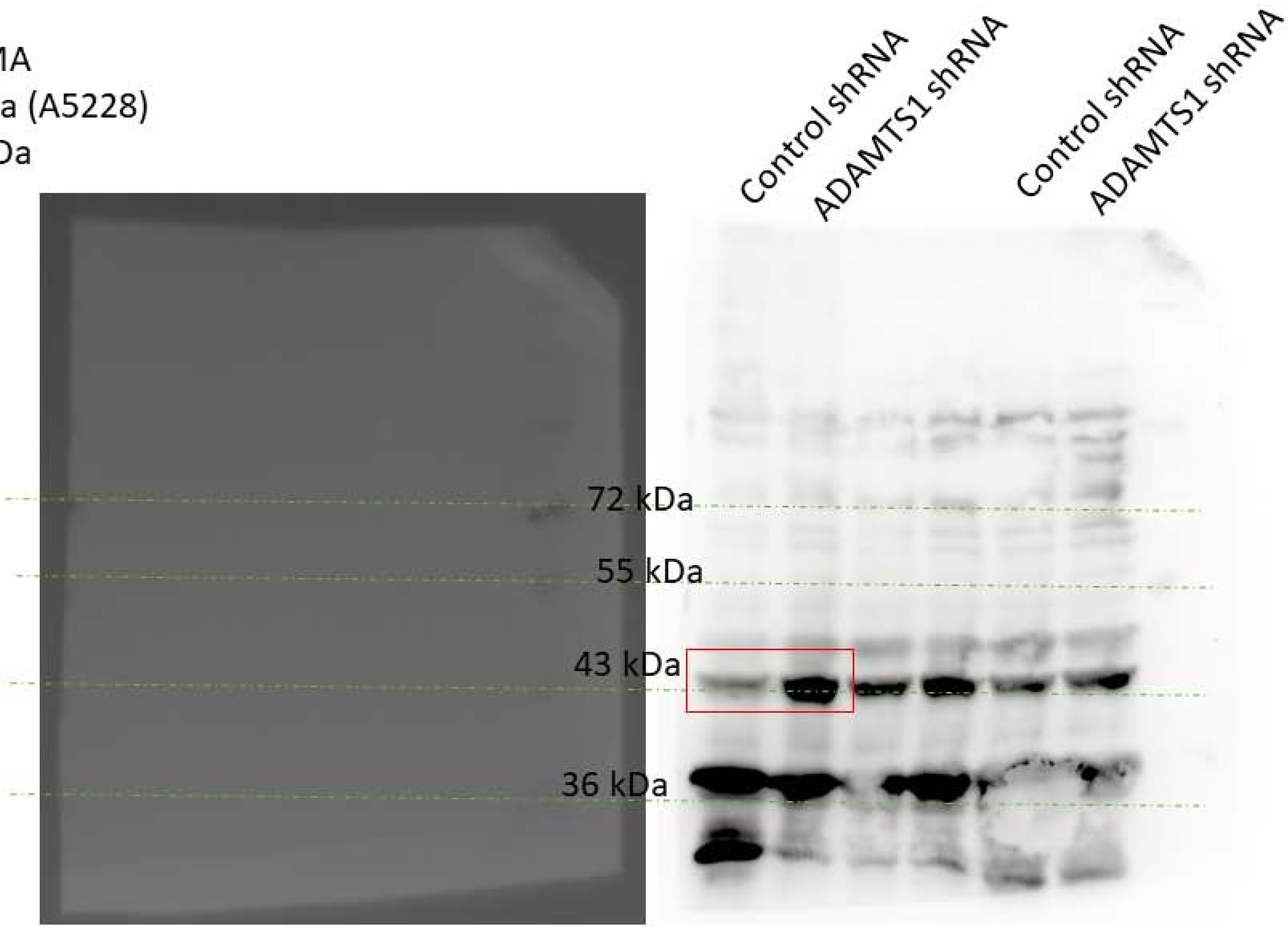

Vimentin
BD (550513)
57 kDa

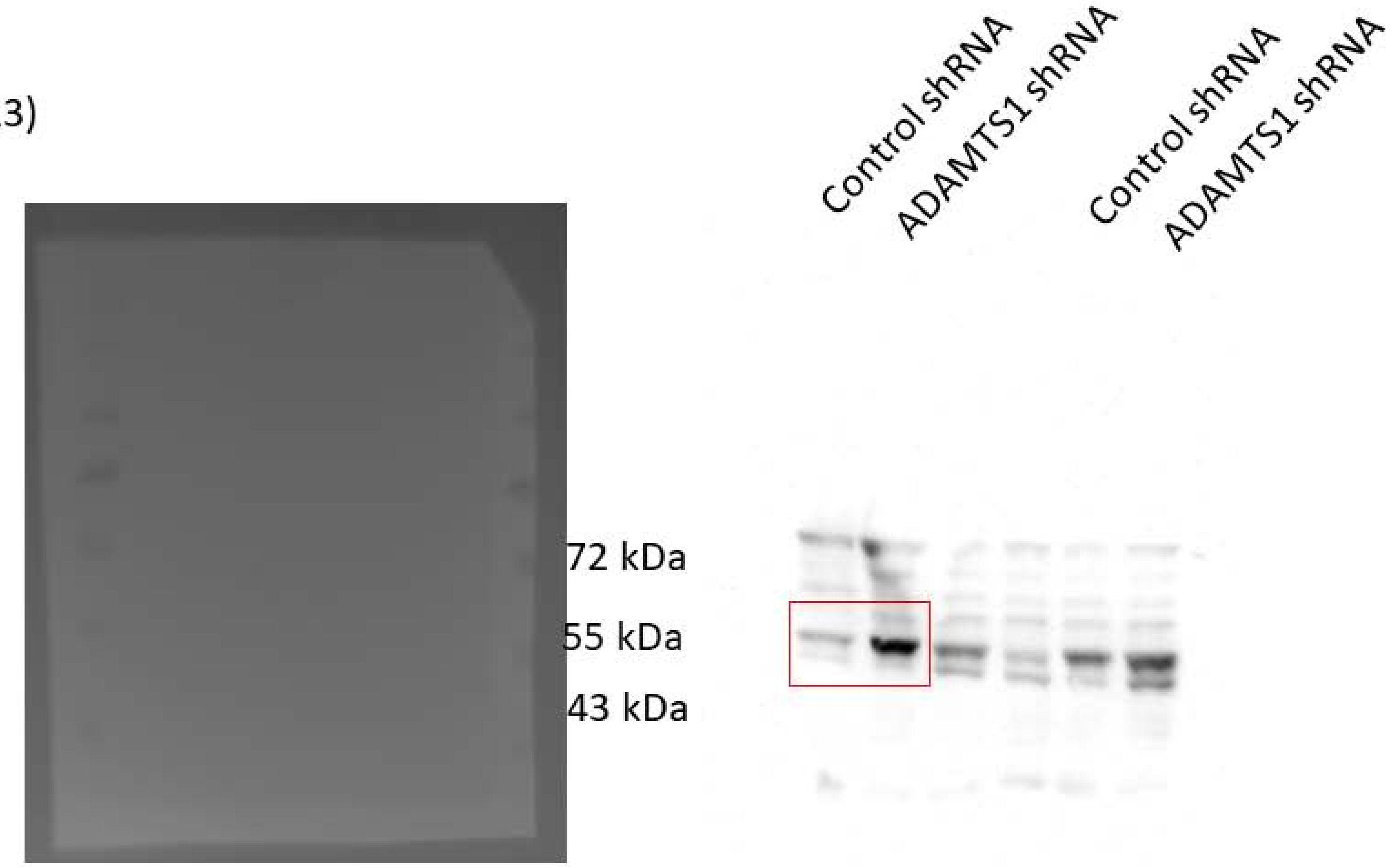

2021.09.25

Snail
Cell Signaling (3879s)
29 kDa

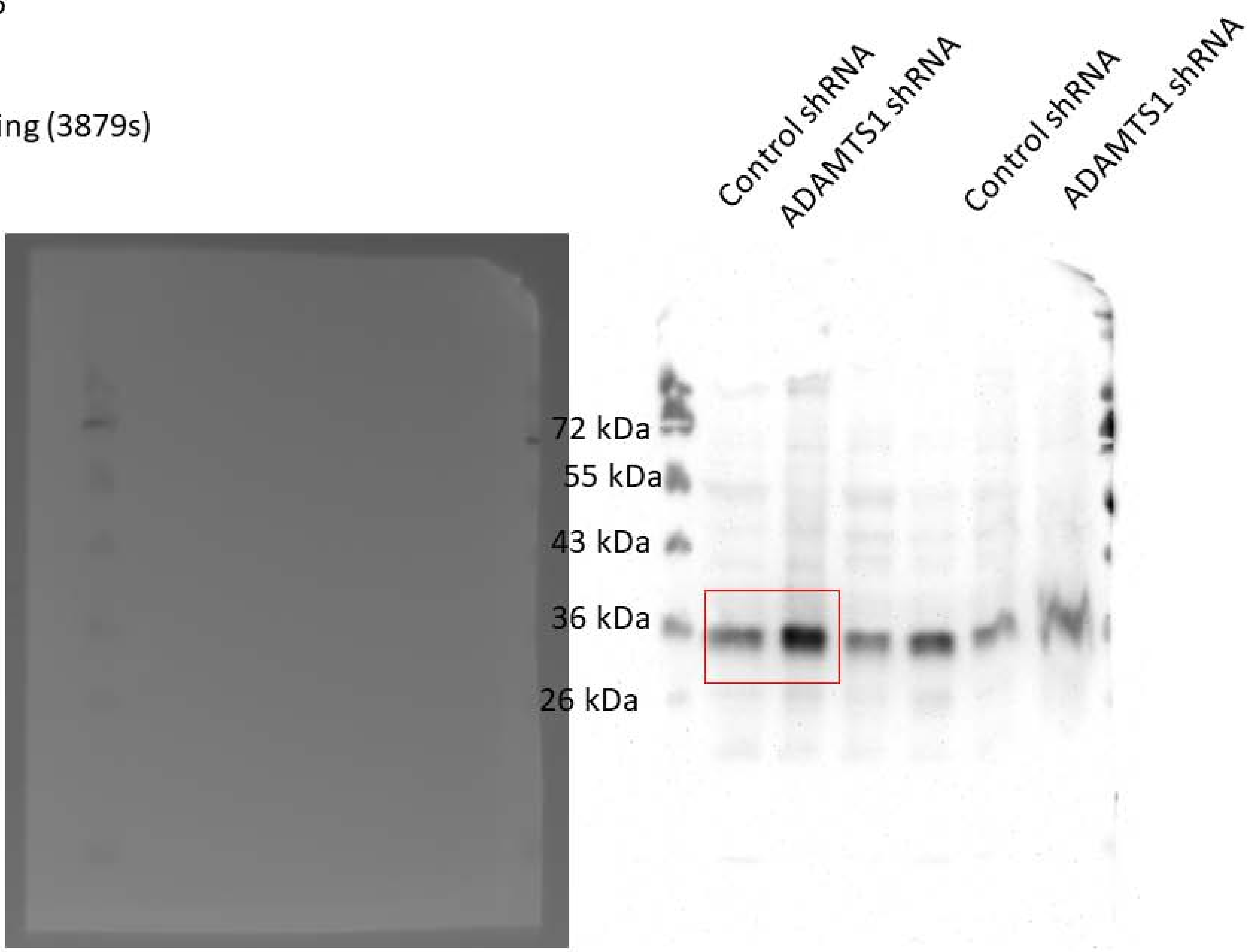

Slug
Cell Signaling (9585s)
30 kDa

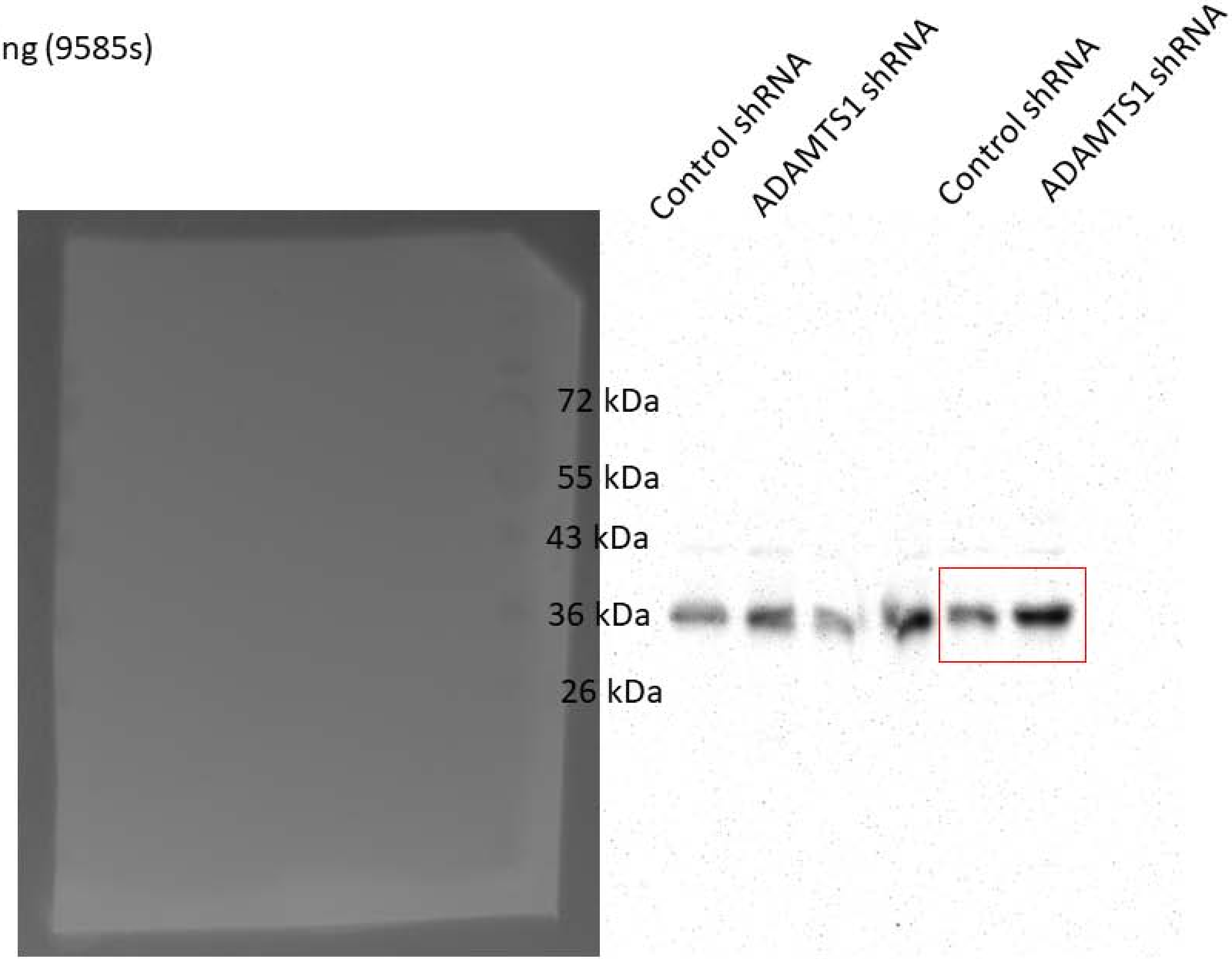

GAPDH
Millipore (MAB374)
38 kDa

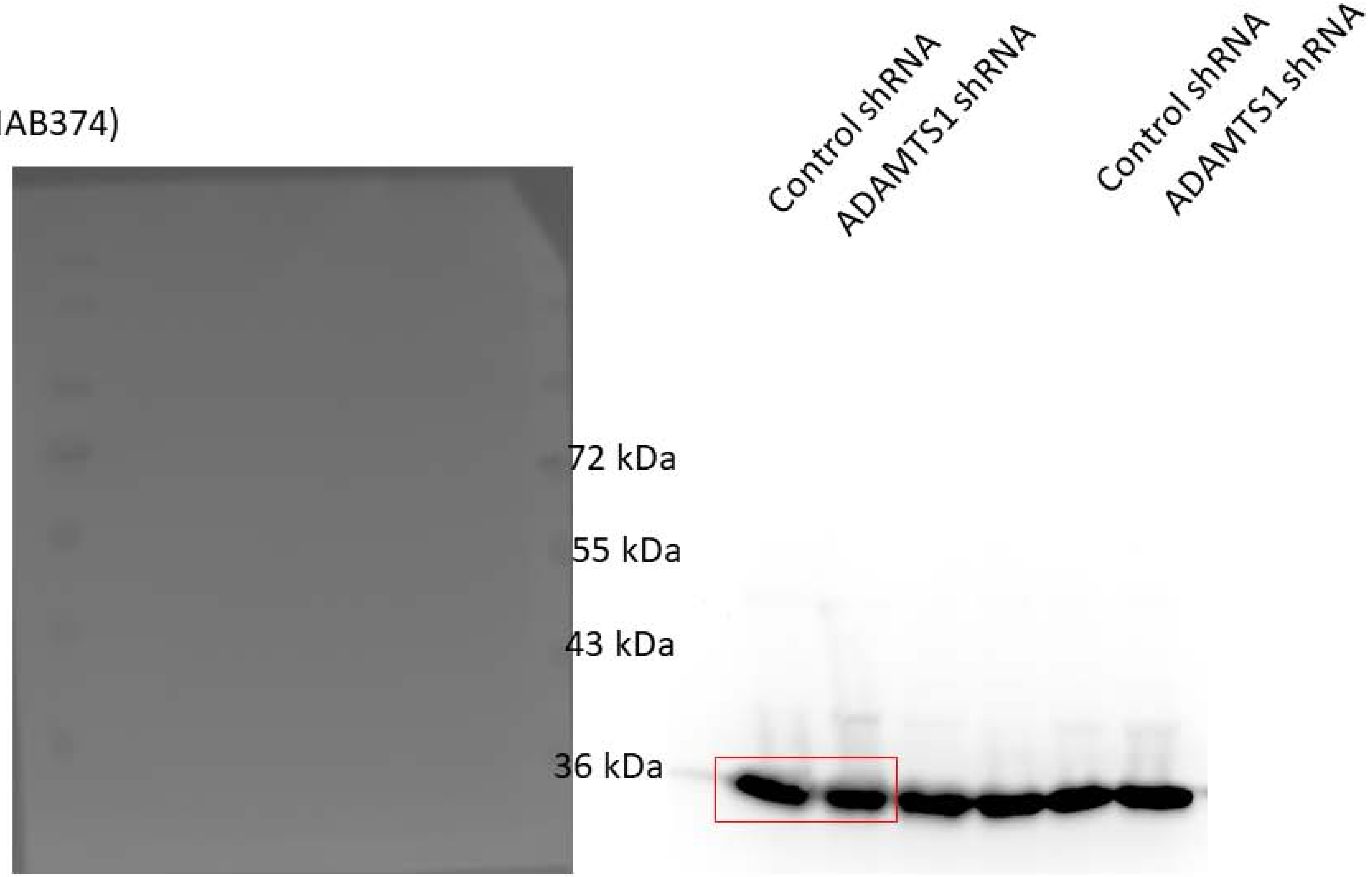

Supplement: Supplementary file 1 [file biology-11-00760-s001.zip › biology-1705610-supplementary.pdf]
